# V-type granular starches derived from different starch varieties: an exploration of the relationships between structure, physicochemical properties, and emulsifiability

**DOI:** 10.1007/s10068-025-01898-9

**Published:** 2025-06-02

**Authors:** Qian Zhou, Qingfei Duan, Huabing Zhai, Fuhan Xie, Yufei Huang, Fengwei Xie, Pei Chen

**Affiliations:** 1 Guangdong Provincial Key Laboratory of Food Quality and Safety, College of Food Science, South China Agricultural University, Guangzhou, 510642 Guangdong China; 2 Institute of Chemistry, Henan Academy of Sciences, Guangzhou, 450002 China; 3 Department of Chemical Engineering, University of Bath, Bath, BA2 7AY UK

**Keywords:** V-type starch, Starch lauric acid complex, Starch emulsifier

## Abstract

V-type starch possesses great potential as a multifunctional emulsifier. However, the relationship between the structure of V-type starch and its emulsifiability remain unclear. Herein, we choose four varieties of starch to prepare ethanol-induced V-type starch (EVS) and ethanol-induced V-type starch-lauric acid complexes (EVS-LA). The emulsions stabilized by EVS-LA exhibited smaller droplet sizes and improved storage stability. Pearson correlation coefficient analysis indicates that emulsifiability displayed a significant positive correlation with contact angle (*r* = 0.69) and a significant negative correlation with D(50) (starch granules) and D(50) (emulsion) (*r* = − 0.71 and − 0.75, respectively). Interestingly, the emulsions stabilized by EVRiS (3 d) and EVRiS-LA (60 d) have identical emulsion droplet sizes but exhibit different storage stabilities. This suggests that the granule size of V-type starch influences short-term emulsifiability, the wettability and granule size of V-type starch influence long-term emulsifiability. This study has enhanced our understanding of V-type starch as a multifunctional emulsifier.

**Graphical abstract:**

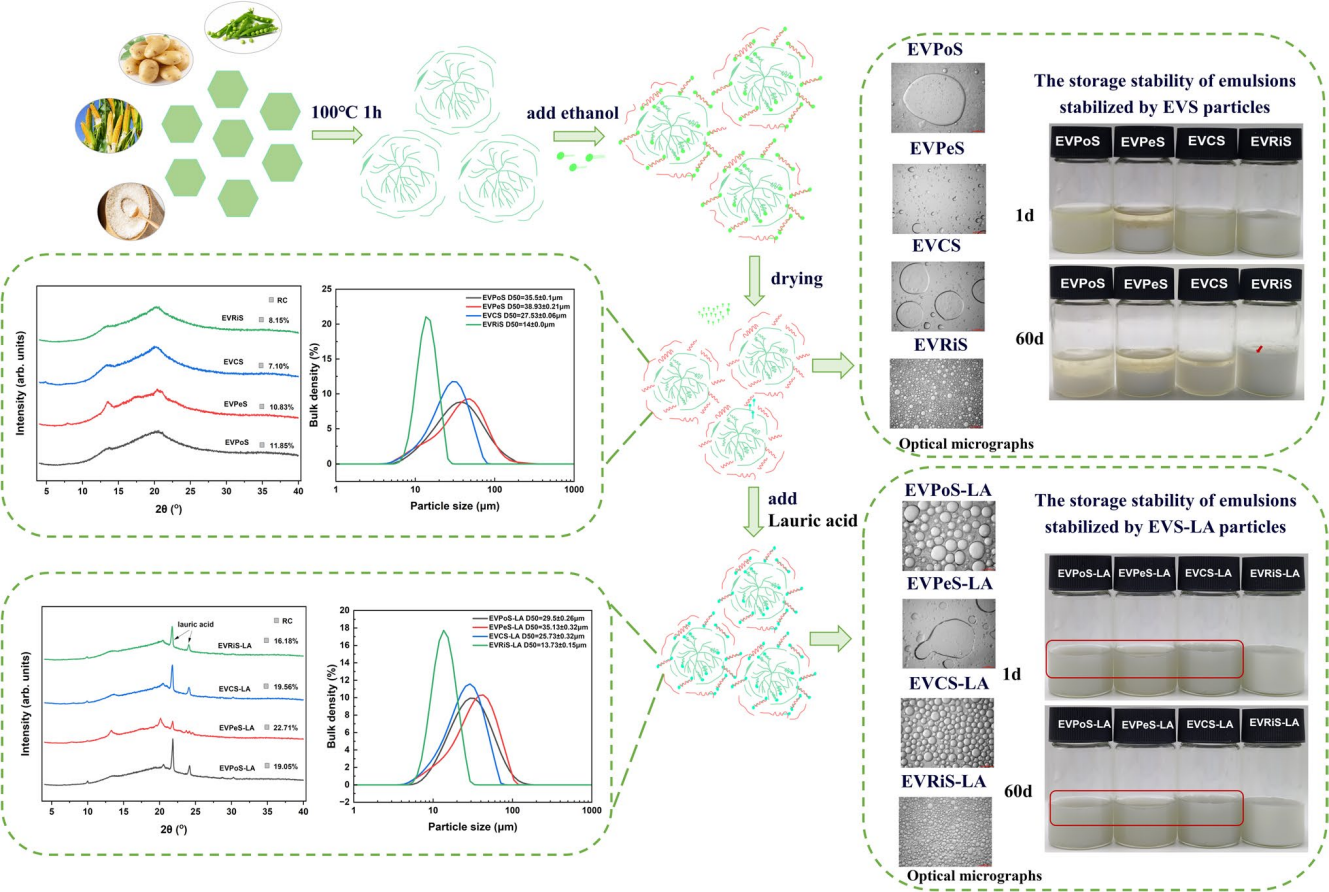

**Supplementary Information:**

The online version contains supplementary material available at 10.1007/s10068-025-01898-9.

## Introduction

Compared to conventional surfactant-stabilized emulsions, Pickering emulsions demonstrate superior performance characteristics, including enhanced kinetic stability and improved compatibility with complex food matrices (Wang et al., 2025). These systems have emerged as important tools in modern food technology, providing unique stabilization mechanisms for a wide range of products such as salad dressings, mayonnaise, coffee creamers, dairy products, margarines, plant-based protein beverages, and ice creams (Tavernier et al., 2016). Recent advances in colloidal science have demonstrated the successful development of edible particulate stabilizers derived from natural biopolymers, particularly proteins and polysaccharides, for effective Pickering emulsion stabilization (Champrasert et al., 2023).

Starch, a ubiquitous natural polymer, is extensively utilized in the formulation of Pickering emulsions owing to its biodegradability, biocompatibility, and affordability (Xu et al., 2020). However, native starch exhibits limited emulsifying capabilities due to its intrinsic hydrophilicity resulting from its polyhydroxy structure. Hence, it becomes crucial to enhance its emulsifying and nutritional properties through modifications (Feng et al., 2022). Previous studies have reported that V-type structured starch obtained through hydrophobic modification exhibits certain emulsifiability (Hedayati et al., 2020; Huang et al., 2021; Li et al., 2024). Recent studies have provided comprehensive investigations into the preparation methodologies, structural configurations, and functional properties of V-type starch. By gelatinizing starch in a boiling water bath, amylose is converted to its destructured (i.e., random coil) state (Kong and Ziegler, 2014). After cooling to a certain temperature, ethanol precipitation induces a transition from ring coiling to helix formation. When ethanol evaporates, a V-type structure with a hydrophobic empty single-helical cavity forms (Liu et al., 2024). This preparation method is a simple and mild physical modification method. Ethanol-induced V-type starch (EVS) possesses notable characteristics, such as higher capacity for cold-water swelling or solubility, making it as a useful food supplement for enhancing food texture. Additionally, EVS has the capability to assemble into complexes and deliver active substances, making it a promising carrier (Li et al., 2024).

The formation of starch-fatty acid complexes is also a method of physical modification. The starch-fatty acid complexes is also of the V-type structure, which is formed primarily between amylose and the hydrophobic fatty acids (Guo et al., 2021). Hydrophobic fatty acids induce the formation of a single helix structure in amylose, which possesses hydrophobic cavities, entering these helical cavities through hydrogen bonding and hydrophobic interactions, ultimately resulting in the formation of starch-fatty acid complexes (Cheng et al., 2018). Current research has confirmed that V-type starch-fatty acid complexes can serve as stabilizers for Pickering emulsions. Lu et al. (2020) first prepared Pickering emulsions using complexes of high-amylose corn starch and lauric acid, and found that these emulsions exhibited good emulsifying ability. Chen et al. (2022) utilized lauric acid to modify high-amylose corn starch during ethanol-based solvothermal treatment, resulting in the preparation of Pickering emulsions, and discovered that variations in pH value and NaCl concentration had a minimal impact on the viscosity and stability of the emulsions.

Research on V-type starch-stabilized Pickering emulsions is still in its infancy, despite its considerable potential as a novel encapsulation matrix for lipophilic bioactive molecules in functional foods and pharmaceutical formulations. Current literature predominantly focuses on model systems utilizing corn starch-fatty acid complexes. The structure–function relationships of V-type starch are governed by: (i) intrinsic molecular features (botanical source, amylose/amylopectin ratio, and amylose chain length distribution), and (ii) extrinsic processing factors, which collectively determine its gelatinization behavior, viscoelastic performance, and emulsification efficacy (Li et al., 2024; Wang et al., 2020). Starches derived from various botanical sources and within-species varieties or lines can exhibit substantial variations in their physicochemical properties (Zhao et al., 2023). However, the impact of starch varieties on the formation and emulsifiability of V-type starch remains unclear. The comprehension of the relationship between the molecular structure of V-type starch and the emulsifiability of Pickering emulsions is still limited.

Therefore, in this work, we employed ethanol precipitation and LA complexation methods to prepare V-type starch using four different starch varieties (corn starch (CS), pea starch (PeS), rice starch (RiS), and potato starch (PoS)), and examined the correlation between V-type starch structure and emulsifiability. This comprehensive research aims to enhance our understanding of V-type starch as a multifunctional emulsifier, with the hope of promoting advancements and development in the dairy and beverage industries.

## Materials and methods

### Materials

Corn starch (12.5% moisture, 85.09 ± 3.64% starch content) was bought from COFCO Haiyou Co., Ltd. (Beijing, China). Potato starch (17.55% moisture, 78.99 ± 1.7% starch content) was bought from Zhuanglang Xinxi Starch Co., Ltd. (Zhuanglang, Gansu). Pea starch (10.14% moisture, 83.49 ± 1.06% starch content) was bought from Shuangta Food Co., Ltd. (Yantai, Shandong). Rice was bought from Shenzhen Shenghao United Grain Co., Ltd. (Shenzhen,China). Soybean oil was purchased from Yihai Grain and Oil Industry Co., Ltd. (Guangzhou, China). Lauric acid (CH_3_(CH_2_)_10_COOH, molecular weight: 200.32) was bought from Hubei Covode Chemical Co., Ltd.

### Preparation of rice starch

Rice was milled with a universal grinding machine and subsequently sieved through a 150 µm screen. A 0.3% (w/v) sodium hydroxide solution was then added to the rice flour in a 1:5 (w/v) ratio, followed by thorough mixing. The mixture was allowed to react on a constant-temperature shaker maintained at 25 °C for 24 h. After the reaction, the pH was adjusted to neutrality. The mixture was centrifuged with a high-speed centrifuge (314.16 rad/s, 600 s). The precipitate underwent multiple centrifugation processes until only the white precipitate remained. After the washing step, the starch precipitate was obtained and subsequently dried at 40 °C for 48 h (Chen et al., 2019). Rice starch (11.68% moisture, 79.55 ± 1.67% starch content).

### Preparation of V-type starch

Different varieties of gelatinized starch were prepared by gelatinizing starch at a concentration of 6% w/v at 100 °C for 1 h. Once the temperature reached 50 °C, the absolute ethanol was added to the starch solution in a ratio of 2:1 (completed within 1 h). After centrifugation, the precipitate was dried in an oven at 40 °C for 12 h and then sieved through a 150 µm screen to obtain V-type starch.

In order to prepare starch-LA complexes, LA with 150 µm screen was mixed evenly with the prepared V-type starch at 10% (w/w), and then placed in a high-temperature cooking bag and subjected to a reaction in an oven at 80 °C for 6 h (Huang et al., 2021). The resulting alcohol precipitate starch samples were labelled as EVS and starch-LA complexes as EVS-LA, while native starches were marked as NS.

### Preparation of Pickering emulsions

Prior to emulsification, suspensions of starch granules were prepared by dissolving them in deionized water at a particle concentration of 6% (w/v). The mixtures were stirred for 4 h to ensure sufficient hydration of starch granules. Subsequently, the starch granule suspensions were mixed with soybean oil at a volume ratio of 5:5.

To create Pickering emulsions, the suspensions of starch granules, soybean oil, and water were combined using a homogenizer fitted with an S25-18D dispersing tool (T18 digital Ultra Turrax, Germany) working at 1361.36 rad/s for 180 s.

## Characterizations of starch-LA complexes

### Amylose content

The amylose content were measured using the method previously outlined by Ge et al. (Ge et al., 2022). The equation was used to determine the amylose content: *y* = 0.0084 *x* + 0.1845, with an *R*^2^ value of 0.9998.

### Complexing index (CI)

The complexing index (CI) values of starch-LA complexes were determined following the methodology previously described by Sun et al. (2023). Using a UV–Vis spectrophotometer (GENESYS 560, Thermo Fisher Scientific Inc., Shanghai, China). The CI values were computed using the provided formula:

1 $$CI\left(\%\right)=\frac{\left({A}_{1}-{A}_{2}\right)\times 100}{{A}_{1}}$$

where A_2_ is the absorbance of the starch-LA complex and A_1_ is the absorbance of the EVS (control).

### Particle size

Prior to measurements, suspensions of starch granules were prepared by dissolving them in deionized water, stirring for 4 h. Afterward, to simulate the state of starch in an emulsion, the suspension was homogenized for 180 s at 1361.36 rad/s using a homogenizer (T18 digital Ultra Turrax, Germany). The laser scattering analyzer (Mastersizer 3000, Malvern, UK) was employed to measure the apparent average size of the samples (Jiang et al., 2023).

### Optical microscopy

Prior to measurements, suspensions of starch granules were prepared by dissolving them in deionized water, stirring for 4 h. Afterward, to simulate the state of starch in an emulsion, the suspension was homogenized for 180 s at 1361.36 rad/s using a homogenizer (T18 digital Ultra Turrax, Germany). The samples were observed using an optical microscope (SMART-POL, Chongqing Aote Optical Instrument Co. Ltd., China). Observations were performed at room temperature in normal light mode. Microscope images were captured with a magnification of 400 × .

### Scanning electron microscopy (SEM)

The samples were imaged using a scanning electron microscope (Hitachi TM4000Plus, Tokyo, Japan) that was set to operate at an acceleration voltage of 15 kV and 1000 × magnification.

### Differential scanning calorimetry (DSC)

The samples were measured using differential scanning calorimeter (DSC 4000, PerkinElmer, Inc., Waltham, Massachusetts, USA) following by Su et al. (2024), with small alterations. Starch sample was suspended in distilled water, maintaining a water-to-complex ratio of 2:1 (w/w, based on dry weight). The suspension was then allowed to equilibrate for 24 h at 4 °C. For DSC measurements, a sample weighing approximately 6 mg (an empty pan served as the control) was scanned from 20 to 150 °C at a heating rate of 10 °C/min.

### X-ray diffractometry (XRD)

The samples were measured using an X-ray diffractometer (Bruker D8 Advance, Bruker AXS Inc., Germany) equipped with Cu-Ka radiation source. The X-ray diffraction analysis was performed with a scanning speed of 2°/min over the range of 4° to 40°. Relative crystallinity (RC) was calculated using Jade 6 software (Lan et al., 2024).

### Fourier-transform infrared (FTIR) spectroscopy

The samples were analyzed using the Nicolet 6700 FTIR spectrometer (Thermo Fisher Scientific, Waltham, MA, USA). The spectrometer was set to scan 64 times within the range of 4000–400 cm^−1^ at a resolution of 4 cm^−1^. The FTIR curves within the wavenumber range of 1200–800 cm^−1^ were deconvoluted using OMNIC software, and the absorbance ratio at 1047 cm^−1^ to that at 1022 cm^−1^ (*R*_1047/1022_) were calculated (Cai et al., 2024b).

### Three-phase contact angle (θ) measurements

The surface wettability of starch granules can be evaluated through contact angle (Du Le et al., 2020; Yao et al., 2023). Before measurement, all samples were pressed between the plates of a mechanical press (SDP-O, Jiangsu Tiancheng Machinery) for 30 s to form a cylindrical tablet. Then, using an ZJ-7000 contact angle measuring device (Zhijia Instruments, China), deionized water (2 µL) was placed onto the sample surface, and an image of the water droplet was captured.

## Characterization of Pickering emulsions stabilized by starch samples

### Emulsions storage stability

The storage stability of Pickering emulsions was assessed by visually examining them in a glass bottle stored at room temperature. The creaming behavior was monitored over a period of 1, 3, 7, 14, 21, 30, 40, 50 and 60 days. The degree of creaming was quantified using emulsification index (EI), determined using the given formula:

2 $$EI\left(\%\right)=\frac{{H}_{e}\times 100}{{H}_{t}}$$

where H_e_ and H_t_ are the height of the emulsification layer and the total height of the emulsion, respectively (Huang et al., 2021).

### Droplet size

The laser scattering analyzer (Mastersizer 3000, Malvern, UK) was employed to measure the apparent average size of the Pickering emulsions. The emulsion was carefully added to the sample cell until the shading rate reached approximately 10–2%. The refractive index (RI) values used for the calculations were 1.47 for Pickering emulsions and 1.33 for water.

### Optical microscopy

To observe the microstructure of Pickering emulsions, a few drops were deposited onto a glass slide within a circular groove, and then covered with a coverslip. An optical microscope (SMART-POL, Chongqing Aote Optical Instrument Co. Ltd., China) was then used to observe the emulsion microstructure, and images were captured with a magnification of 200 × (Cai et al., 2024a).

### Rheological behavior

The Pickering emulsions with different samples were analyzed using a rheometer (MCR 502, Anton Paar, Germany). The rheometer containing stainless steel parallel plates (plate diameter of 25 mm, gap of 1 mm) (Wang et al., 2024). The viscoelastic properties of the samples were evaluated through dynamic frequency sweeping with an angular frequency range of 0.1 to 10 rad/s at a 1% strain. Furthermore, steady shear tests were performed within a shear rate range of 0.01 to 100 s^−1^ on the samples to determine their apparent viscosity.

### Statistical analysis

All data are presented as mean ± standard deviation (*n* = 3). The significant differences among these mean values were evaluated through one-way Analysis of Variance (ANOVA). The statistical significance was set at *p* < 0.05.

## Results and discussion

### Amylose content

**Fig. 1:**
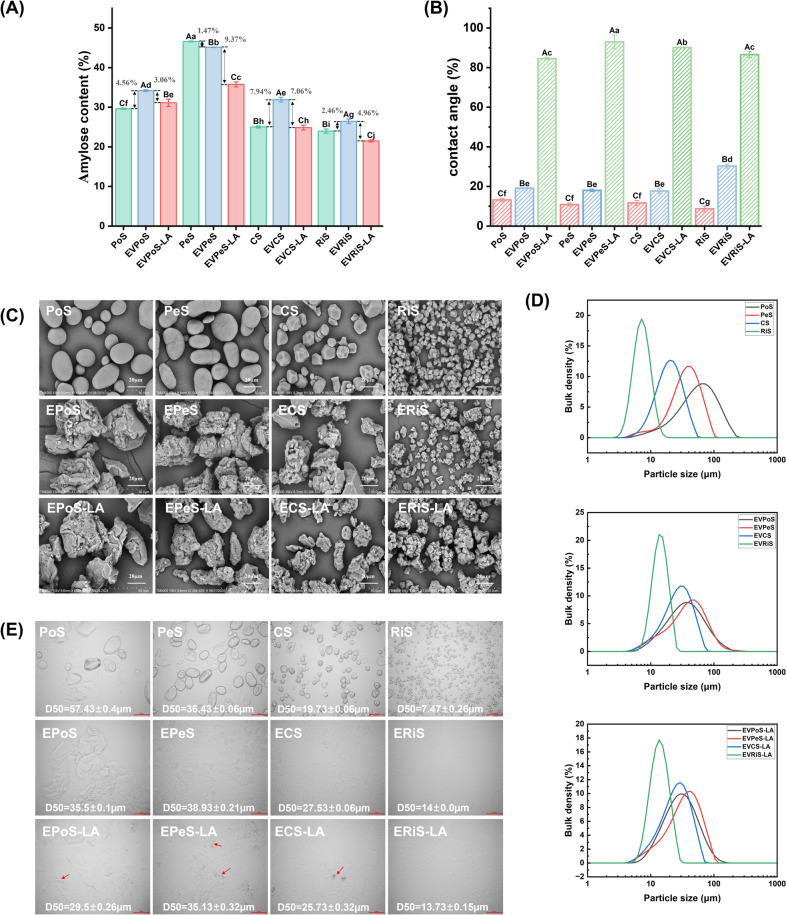
**A** Amylose content data of NS, EVS, and EVS-LA, **B** contact angle values of NS, EVS, and EVS-LA, **C** SEM micrographs of NS, EVS, and EVS-LA, **D** particle size distribution of NS (a), EVS (b), and EVS-LA (c) and **E** Light-microscopic images of NS, EVS, and EVS-LA. Different uppercases (A–C) and lowercases (a–i) suggest significant differences (*p* < 0.05)

The amylose content of the NS, EVS, and EVS-LA samples obtained using the iodine binding method are displayed in Fig. 1A. Compared to NS, the amylose content of starches significantly increased following gelatinization and ethanol precipitation, except for EVPeS. This phenomenon could be attributed to the swelling of starch granules and subsequent leaching of amylose during heating in excess water, combined with the partial degradation of amylopectin during gelatinization, both of which lead to an increase in amylose content (Zhu, 2015). Upon complexation between EVS and LA, the apparent amylose content decreases significantly. This reduction results from the formation of complexes between amylose and fatty acids (Le et al., 2018). Fatty acids have an affinity for binding with amylose helices, which in turn decreases the accessibility of unbound amylose sites for interaction with iodine. Consequently, fewer available amylose binding sites result in a weaker color intensity of the amylose-iodine complex for amylose quantification (Thakur et al., 2017).

### Complexing index (CI)

The CI values of different starch-LA complexes formed with different starch varieties are illustrated in Table 1. A greater CI value indicates a more extensive complexation between amylose and LA (Chao et al., 2020). Compared to other complexes, EVPeS-LA and EVCS-LA exhibited higher CI values (*p* < 0.05). The EVPoS-LA complex exhibited the lowest CI value. This could stem from the higher amylose content in EVPeS (45.14 ± 0.07%) compared to other complexes (Fig. 1A and Table S1). Starch that contains a high level of amylose can accommodate more LA molecules to bind with its helical structure, thereby increasing the production of amylose-LA complexes (Sun et al., 2023). As shown in Fig. 1A and Table S1, potato starch exhibits a relatively high amylose content and a high amylose/amylopectin ratio. However, the EVPoS-LA complex displays the lowest CI value, which may be associated with the proportion of short chains in amylopectin. The formation of starch-fatty acid complexes relies on the long side chains of both amylose and amylopectin. Starch-fatty acid complexes formation may be impacted by the shorter chain lengths of amylopectin branches and their steric hindrance (He et al., 2020; Wang et al., 2016). Previous work has shown that the side chain length distribution of short chains in different amylopectin sources (Chung and Liu, 2009). Alternatively, the high viscosity of potato starch may inhibit the formation of complexes. The CI value aligns with the decrease in amylose content observed in EVS as discussed above.

**Table 1:** Complex index (CI) values of different EVS-LA complexes

Sample	CI
EVPoS-LA	86.77 ± 0.97^c^
EVPeS-LA	91.27 ± 0.21^a^
EVCS-LA	90.5 ± 0.3^a^
EVRiS-LA	88.4 ± 0.36^b^

Different lowercase letters (a-c) indicate significant differences (*p* < 0.05)

### Three-phase contact angle

Figure 1B demonstrates that native starch samples exhibit strong hydrophilic characteristics, with contact angles measuring 13.21° (PoS), 10.88° (PeS), 11.67° (CS), and 8.75° (RiS). These low contact angle values confirm the pronounced surface hydrophilicity typical of hydrophilic colloids. The intense hydrophilicity of native starch is attributed to hydrogen bonding between the excessive free hydroxyls on starch surfaces with water (Apostolidis et al., 2024; Ge et al., 2017). After alcohol precipitation, the contact angles of EVPoS, EVPeS, EVCS, and EVRiS increased to 19.06°, 18.06°, 17.72°, and 30.28°, respectively, indicating an enhancement in their hydrophobicity. Ethanol can induce amylose to form a V-type single helical structure, and after evaporation, a hydrophobic cavity is formed (Li et al., 2024).

When complexed with LA, the contact angles of EVPoS-LA, EVPeS-LA, EVCS-LA, and EVRiS-LA further increased to 84.6°, 93°, 90.12°, and 86.56°, respectively. The V-type single helical structure could interact with fatty acid molecules, forming new complexes that exhibit enhanced hydrophobic properties compared to EVS (Huang et al., 2021; Liu et al., 2024). Significant differences exist in the contact angle among different EVS-LA complexes, with a sequence of EVPeS-LA > EVCS-LA > EVRiS-LA > EVPoS-LA, which aligns with the CI results. These variations may result from differences in particle size, amylose content, and average side chain length of amylopectin among different starches. These differences ultimately affect the distribution of LA covering the outside of starch granules, amorphous lamellae, and crystalline lamellae (He et al., 2020).

### Comparison of particle size and morphological characteristics among different samples

Figure 1C depicts the morphological characteristics of the NS, EVS, and EVS-LA samples examined through SEM. The different starch varieties exhibited variations in size and shape. Native CS granules and native RiS granules exhibited circular or polygonal irregular shapes, while native PoS granules had smooth surfaces and were elliptical or spherical in shape. In contrast, native PeS granules were primarily discoidal in shape and had concave faces. However, after gelatinization and ethanol precipitation, EVPoS, EVPeS, and EVCS granules underwent significant morphological transformations. These granules were destroyed, melted, and formed agglomerates, resulting in debris-shaped granules. EVRiS granules showed wrinkles and fissures on their surfaces, with some granules melting and sticking together. These granules showed mainly a rough surface. When these granules were combined with LA, they also exhibit predominantly rough surfaces and agglomeration. According to previous research (Marinopoulou et al., 2016), amylose-fatty acid complexes exhibit a lamellar morphology, with their rough surfaces potentially due to amylose-fatty acid complexes. The agglomeration phenomenon was due to both alcohol precipitation and the formation of amylose-lauric acid complexes.

The light microscopy images and particle size parameters of NS, EVS, and EVS-LA are shown in Fig. 1D and E. The figures clearly showed that all granules have become more uniform, displaying a unimodal distribution. In comparison with NS, the average granule size of EVS was 1.2 to 3 times larger (*p* < 0.05), which is consistent with previous research findings (Zhou et al., 2021). EVS was prepared by subjecting native starch to gelatinization and subsequent alcohol precipitation. During ethanol evaporation, the single helices of amylose and amylopectin form large cavities, leading to water absorption and swelling at room temperature (Majzoobi and Farahnaky, 2021). The light microscopy images also clearly show an increase in EVS granule size, indicating enhanced swelling and water-absorption capabilities.

After being combined with LA, the average granule size of EVS-LA was smaller than that of EVS. This decrease in size may be attributed to increased hydrophobic interactions within EVS caused by the addition of LA, which hinder water absorption and swelling capabilities. Previously, starch-fatty acid complexes have been mentioned that appear as spherical and filamentous granules (Lu et al., 2019). The starch-LA complexes are observable in Fig. 1C, displaying similar characteristics.

### DSC analysis

Table S2 presents the thermal properties of NS and EVS samples. The gelatinization temperature of the four NS samples ranged from 60.4 to 77.25 °C (PoS), 60.27 to 74.23 °C (PeS), 65.32 to 79.57 °C (CS), and 69.64 to 84.71 °C (RiS), respectively, which aligns with previous studies (Man et al., 2012; Ouyang et al., 2021). After undergoing alcohol precipitation, the gelatinization temperatures of all EVS samples were significantly reduced, generally falling within the range of 46.25–75.23 °C. This indicates that the granules of EVS are more prone to gelatinization after alcohol precipitation.

In addition, the Δ*H* values of EVS samples after alcohol precipitation (4.31–7.42 J/g) was lower than those of NS samples (8.66–25.51 J/g). Gelatinization disrupts the structure of starch, generating a significant quantity of linear chains. When these linear chains undergo alcohol precipitation, they reassemble to form single-helical complexes, resulting in a novel crystalline structure characterized by a V-type configuration. However, the V-type crystalline structure prepared by this method can be easily destroyed (Deng et al., 2023).

Table S3 presents the thermal properties of the different EVS-LA complexes. These complexes displayed three distinct endotherms (Table S3). The first endotherm (Peak I), observed within the temperature range of 42–50 °C, stems from the melting of unbound LA (Liu et al., 2016). The second (Peak II) and third peak (Peak III) endotherms, observed at higher temperatures ranging from 88.65 to 137.83 °C, correspond to the melting of V_I_-type (starch (host)-guest structure, or inclusion complex) and V_II_-type (lamellae-like semi-crystalline structure) amylose-LA complexes (Lu et al., 2021; Zhang et al., 2012; Zhou et al., 2022).

The Δ*H* associated with amylose-LA complexes serves as an indicator for both the degree of complex formation and the internal order within the complex. The influence of the amount of complex formation on Δ*H* is greater than that of the internal order (Wang et al., 2016). As indicated in Table S3, the EVPeS-LA complex exhibited the highest Δ*H* at the third endotherm (peak III), indicating a higher formation of the V_II_-type complex. Furthermore, the EVCS-LA complex displayed the highest Δ*H* at the second endotherm (peak II), indicating a higher production of the V_I_-type complex. The total Δ*H* of the second (Peak II) and third (Peak III) endotherms reflects the quantity of a complex generated between LA and amylose, following the order of EVPeS-LA > EVCS-LA > EVRiS-LA > EVPoS-LA. The findings align with the results obtained from CI and contact angle measurements (Table 1 and Fig. 1B).

### XRD analysis

**Fig. 2:**
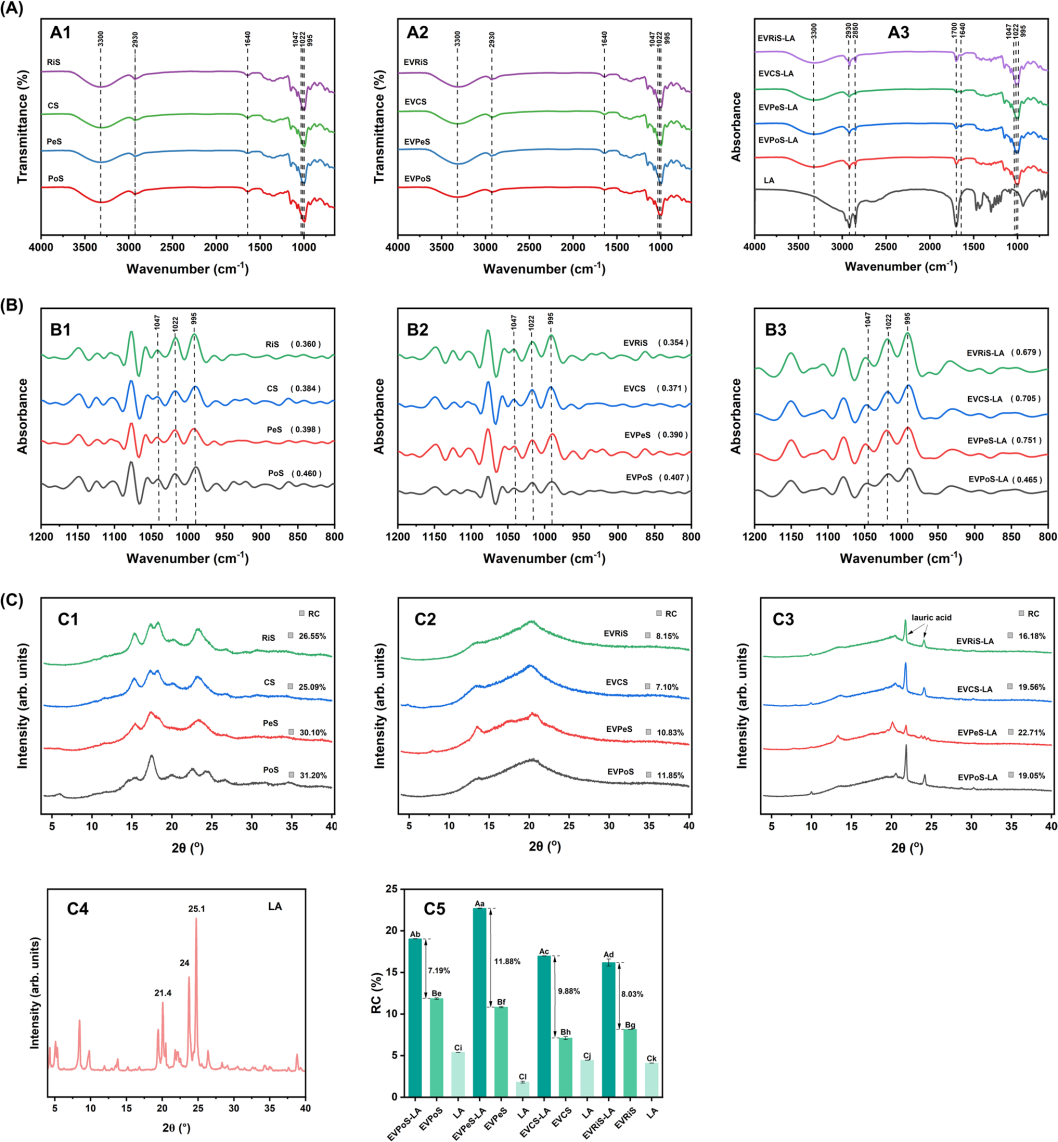
**A** FTIR spectra of NS, EVS, and EVS-LA. NS, EVS, and EVS-LA samples, **B** enlarged spectra within the range of 1200–800 cm^−1^ and **C** XRD patterns and relative crystalline values of NS, EVS, EVS-LA, LA samples

Figure 2C shown the XRD patterns of the NS, EVS, and EVS-LA samples. CS and RiS samples exhibited the typical A-type crystalline, displaying reflections at approximate 2*θ* values of 15.3° and 23.3°, accompanied by double diffraction peaks at 17.2° and 18.1°. PoS displayed a prominent diffraction peak at 17.2°, with smaller peaks approximately 5°, 22°, and 24.2°, indicating the characteristic features of the B-type diffraction pattern. PeS exhibited reflections at 2*θ* values of 17.3° and 23.4°, indicative of the typical C-type diffraction pattern. These findings align with previous studies (Gonzalez and Wang, 2023).

Following alcohol precipitation, all of the EVS samples showed primary diffraction peaks around 7°, 13°, and 20°, indicating the V-type crystalline structure (Li et al., 2022). When complexed with LA, the EVS-LA complexes exhibited a prominent V-type crystalline structure, with major diffraction peaks at 7.5°, 13°, and 20°. Compared to EVS, the diffraction peaks of EVS-LA were notably sharper and higher, indicating the development of single-helix inclusion complexes between the guest molecules and starch (Liu et al., 2020). Although both EVS and EVS-LA predominantly exhibited the V-type crystalline structure, it is evident that the intensity of the intensity of the diffraction peaks and the peak widths narrowed after complexation with LA (Fig. 2C2 and C3). In addition, LA exhibits characteristic peaks at 21.4°, 24.0°, and 25° (2*θ*) (Fig. 2C4), while the peaks at 21.4° and 24.0° (2*θ*) in Fig. 2C3 correspond to the crystalline form of free LA. Kang et al. (2022a) shows that lauric acid exhibits characteristic peaks at 21.4° and 24.0° (2*θ*).

Figure 2C5 shows the RC results of the EVS, EVRiS-LA and free LA. As shown in Fig. 2C5, the RC of EVS-LA is higher than that of EVS, as it includes contributions from both EVS and LA-complex formation. The difference in RC between EVS-LA and EVS reflects the strength of the interactions between EVS and LA. EVPeS-LA exhibits the highest RC (Fig. 2C5), with the largest RC difference between EVPeS-LA and EVPeS (11.88%). This indicates stronger EVPeS-LA interactions compared to other starches, resulting in complexes with enhanced long-range structural order. The RC difference between EVS-LA and EVS shows the order EVPeS-LA > EVCS-LA > EVRiS-LA > EVPoS-LA, reflecting the same order in complex formation, which aligns with the CI and DSC results.

### FTIR analysis

Figure 2A shown the FTIR spectra of NS, EVS, and EVS-LA, providing valuable information regarding the composition and structural alterations of the samples. The bond at 3300 cm^−1^ results from the stretching vibration of hydroxyl groups (–OH), while the bond at 2930 cm^−1^ is associated with the asymmetric stretching of –CH groups. The band at 1640 cm^−1^ is indicative of the O–H stretching vibration of adsorbed water (Liu et al., 2023). EVS displayed identical absorption peaks to NS at corresponding positions, suggesting that no novel chemical bonds were generated during the ethanol treatment process.

However, in comparison to EVS, EVS-LA samples present new characteristic peaks at 2850 and 1700 cm^−1^. These peaks correspond to the asymmetric C–H stretching and C**=**O stretching vibrations of a small amount free LA, respectively (Kang et al., 2022a). The bands at 2850 and 1700 cm^−1^ exhibited an intensity sequence of EVPoS-LA > EVRiS-LA > EVCS-LA > EVPeS-LA among the samples, aligning with the CI and DSC results. FTIR signals are independent of sample weight but are related to sample concentration (Chen et al., 2018). The LA signal intensity directly related to its concentration. This observation may be due to the differing shorter chain lengths of amylopectin branches from different starch sources. When the LA content was fixed at 10.0%, the shorter chain lengths of amylopectin branches and their steric hindrance can affect the formation of starch-fatty acid complexes (Gutiérrez and Bello-Pérez, 2022).

Figure 2B displays the deconvoluted spectra of the samples. The intensity ratio at 1047/1022 cm^−1^ can provide insights into the degree of short-range ordering (Kang et al., 2022b). Upon gelatinization and alcohol precipitation, the intensity ratio decreased, suggesting a diminishing of the short-range ordering of EVS, which was potentially due to the detrimental effects of high temperatures on the starch structure. However, with the addition of LA, the ratio increased, which indicate the development of a more ordered structure. As show in Fig. 2B, the EVPeS-LA sample exhibited a significantly higher ratio compared to other starch-lipid complexes, with the following descending order: EVPeS-LA > EVCS-LA > EVRiS-LA > EVPoS-LA. These results were in agreement with the structural and thermal characteristics revealed by XRD, DSC, and CI analyses.

### Morphology and droplet sizes analysis

**Fig. 3:**
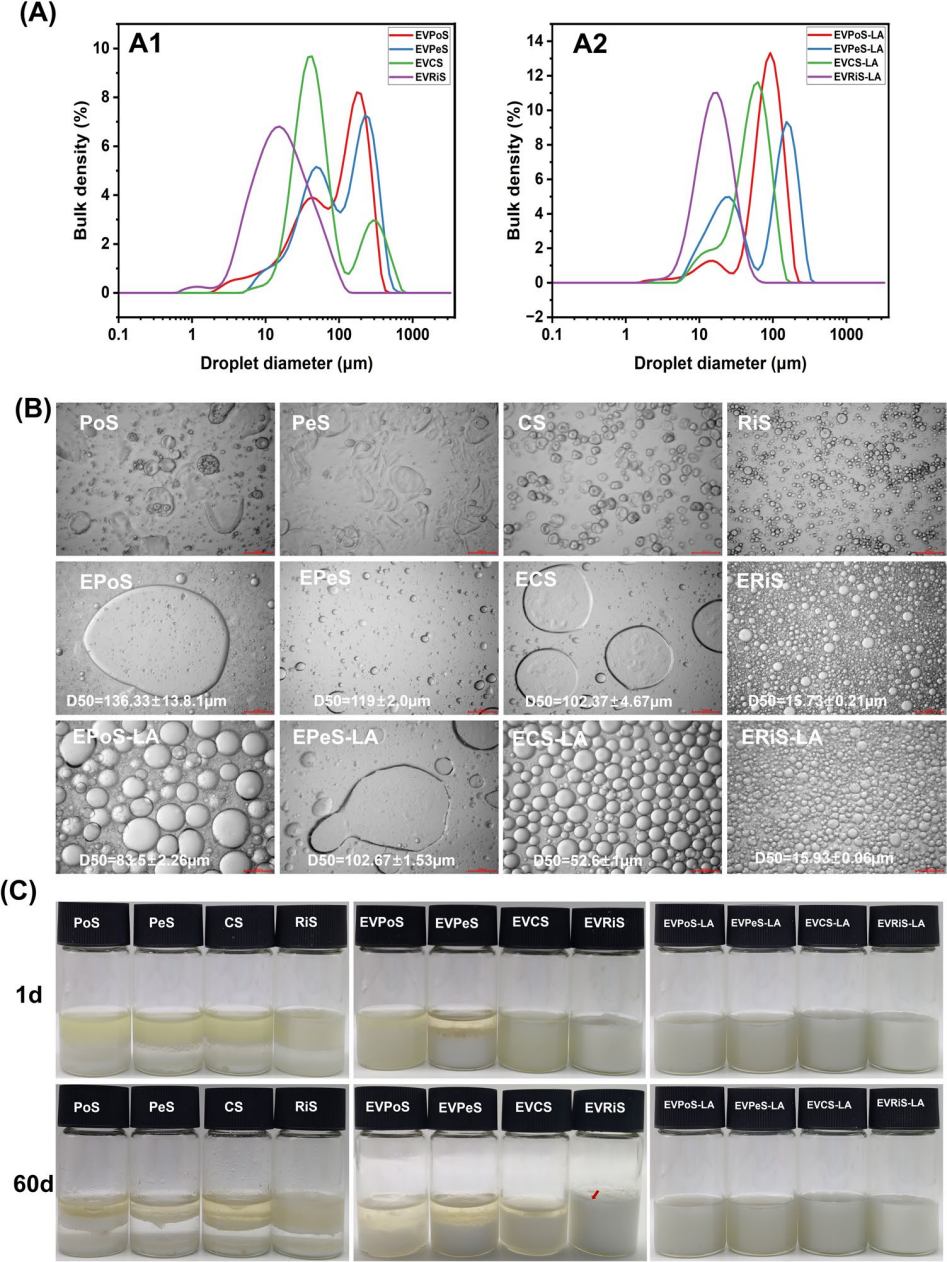
**A** Droplet size distribution curves of Pickering emulsions stabilized by EVS and EVS-LA, **B** light-microscopic images of Pickering emulsions stabilized by NS, EVS, and EVS-LA and **C** photographs of freshly prepared Pickering emulsions and those stored for 60 days

Figure 3A and B show the droplet size distributions and optical characteristics of fresh emulsions. The high hydrophilicity of NS partially limited its emulsifying capability, making the emulsion droplets invisible. With the use of EVS, emulsion droplets became visible, and the droplet size followed this sequence: EVRiS > EVCS > EVPeS > EVPoS. It is noteworthy that all emulsions exhibit a bimodal pattern in their droplet size distributions.

With the use of EVS-LA, the emulsion droplets underwent a transformation, shifting from being large and inhomogeneous to being small and uniform. In this case, the emulsion droplet particle size followed the sequence of EVRiS-LA > EVCS-LA > EVPoS-LA > EVPeS-LA (Fig. 3B). Except for the emulsion formed with EVRiS-LA, the particle size distribution in other emulsions showed multiple peaks. For EVPoS, EVPeS, EVCS, and EVPeS-LA, two prominent peaks were observed, suggesting that these emulsions were less stable compared to others and may have been undergoing slow flocculation or recoalescence. It is noteworthy that the main peak of EVRiS, EVCS-LA, and EVPoS-LA exhibited a small shoulder to the left, likely due to non-adsorbed starch granules.

With the exception of rice starch, the modification methods employed on starches of the same variety exhibited a progressive shift of the main particle size peak towards smaller droplet size as the contact angle increased. This correlation between droplet size and contact angle suggests that a higher contact angle value, indicative of greater wettability to droplets on the oil–water interface, leads to smaller droplet sizes (Champrasert et al., 2023).

Furthermore, when considering different starch varieties, the size of starch granules also influenced emulsion droplet size. The smaller size of starch granules led to the formation of smaller emulsion droplets in the dispersed phase, thereby increasing the surface area covered per unit mass, consistent with previous research findings (Apostolidis et al., 2023; Ge et al., 2017).

### Emulsifiability and storage stability analysis

Table 2 presents the EI, a key indicator used to evaluate emulsifying capability (Zhang et al., 2021), for NS, EVS, and EVS-LA-stabilized Pickering emulsions over a 60-day storage period. The EI value of NS was limited partially due to its high hydrophilicity. However, after alcohol precipitation, the EI of EVS significantly increased (*p* < 0.05), with the emulsifiability following the sequence: EVRiS > EVCS > EVPoS > EVPeS. After starch was complexed with LA, the EI values of all samples except EVRiS-LA were increased (*p* < 0.05), and the emulsifiability followed the sequence: EVRiS-LA > EVCS-LA > EVPoS-LA > EVPeS-LA. All the samples show that the EI value gradually decreases as the storage time extends. Additionally, Fig. 3C displays photographs of emulsions at different storage times (1 and 60 days). Comparing the emulsions of NS, EVS, and EVS-LA, significant improved emulsion stability can be observed. The emulsifiability was clearly enhanced with EVS and EVS-LA.

**Table 2:** Emulsifying capacity of Pickering emulsion stabilized by NS, EVS, and EVS-LA

Samples	EI value								
	1 d	3 d	7 d	14 d	21 d	30 d	40 d	50 d	60 d
PoS	13.83 ± 0.6^ g^	11.8 ± 1.3^e^	9.57 ± 1.94^ g^	0 ± 0^ h^	0 ± 0^ h^	0 ± 0^ g^	0 ± 0^ g^	0 ± 0^ g^	0 ± 0^ g^
PeS	1.67 ± 0.51^i^	0 ± 0^ h^	0 ± 0^j^	0 ± 0^ h^	0 ± 0^ h^	0 ± 0^ g^	0 ± 0^ g^	0 ± 0^ g^	0 ± 0^ g^
CS	3.6 ± 1.1^ h^	3.5 ± 2.23^ g^	3.2 ± 2.13^i^	0 ± 0^ h^	0 ± 0^ h^	0 ± 0^ g^	0 ± 0^ g^	0 ± 0^ g^	0 ± 0^ g^
RiS	32.43 ± 1.43^f^	7.6 ± 3.04^f^	6.93 ± 0.85^ h^	6.57 ± 1.48^ g^	6.5 ± 1.51^ g^	0 ± 0^ g^	0 ± 0^ g^	0 ± 0^ g^	0 ± 0^ g^
EVPoS	58.23 ± 1.15^d^	56.4 ± 0.82^c^	54.67 ± 1.4^e^	54.83 ± 0.21^e^	53.47 ± 0.5^e^	51 ± 0.75^e^	47.5 ± 1.97^e^	48.27 ± 1.04^e^	47.93 ± 1.32^e^
EVPeS	51.17 ± 0.85^e^	46.7 ± 1.11^d^	44.73 ± 0.81^f^	48.3 ± 2.3^f^	45.43 ± 2.57^f^	43.33 ± 4.22^f^	43.9 ± 3.75^f^	41.83 ± 3.52^f^	43.63 ± 0.85^f^
EVCS	93.37 ± 0.75^c^	93.63 ± 0.76^b^	91.2 ± 0.89^d^	90.53 ± 1.3^d^	87 ± 0.85^d^	88.57 ± 0.45^d^	89.03 ± 0.8^d^	87.37 ± 1.29^d^	86.9 ± 1.81^d^
EVRiS	100 ± 0^a^	100 ± 0^a^	94.6 ± 1.14^bc^	92.67 ± 0.6^c^	91.37 ± 1.27^c^	91.5 ± 0.8^c^	91.5 ± 0.92^bc^	92.17 ± 1.48^c^	91.6 ± 0.36^c^
EVPoS-LA	100 ± 0^a^	98.13 ± 0.59^a^	96.17 ± 0.51^b^	95.8 ± 1.01^b^	94.57 ± 0.75^b^	94.8 ± 0.36^b^	93.03 ± 0.78^b^	93.73 ± 0.5^c^	92.5 ± 1.2^c^
EVPeS-LA	96.5 ± 1.31^b^	95.23 ± 1.1^b^	93.37 ± 0.7^c^	92.83 ± 0.97^c^	91.77 ± 0.45^c^	91.77 ± 0.75^c^	90.47 ± 0.57^ cd^	89.43 ± 2.17^d^	88.27 ± 1.78^d^
EVCS-LA	100 ± 0^a^	100 ± 0^a^	100 ± 0^a^	100 ± 0^a^	100 ± 0^a^	100 ± 0^a^	100 ± 0^a^	96.63 ± 1.53^b^	96.37 ± 0.76^b^
EVRiS-LA	100 ± 0^a^	100 ± 0^a^	100 ± 0^a^	100 ± 0^a^	100 ± 0^a^	100 ± 0^a^	100 ± 0^a^	100 ± 0^a^	100 ± 0^a^

Different lowercase letters (a–i) indicate significant differences (*p* < 0.05)

**Fig. 4:**
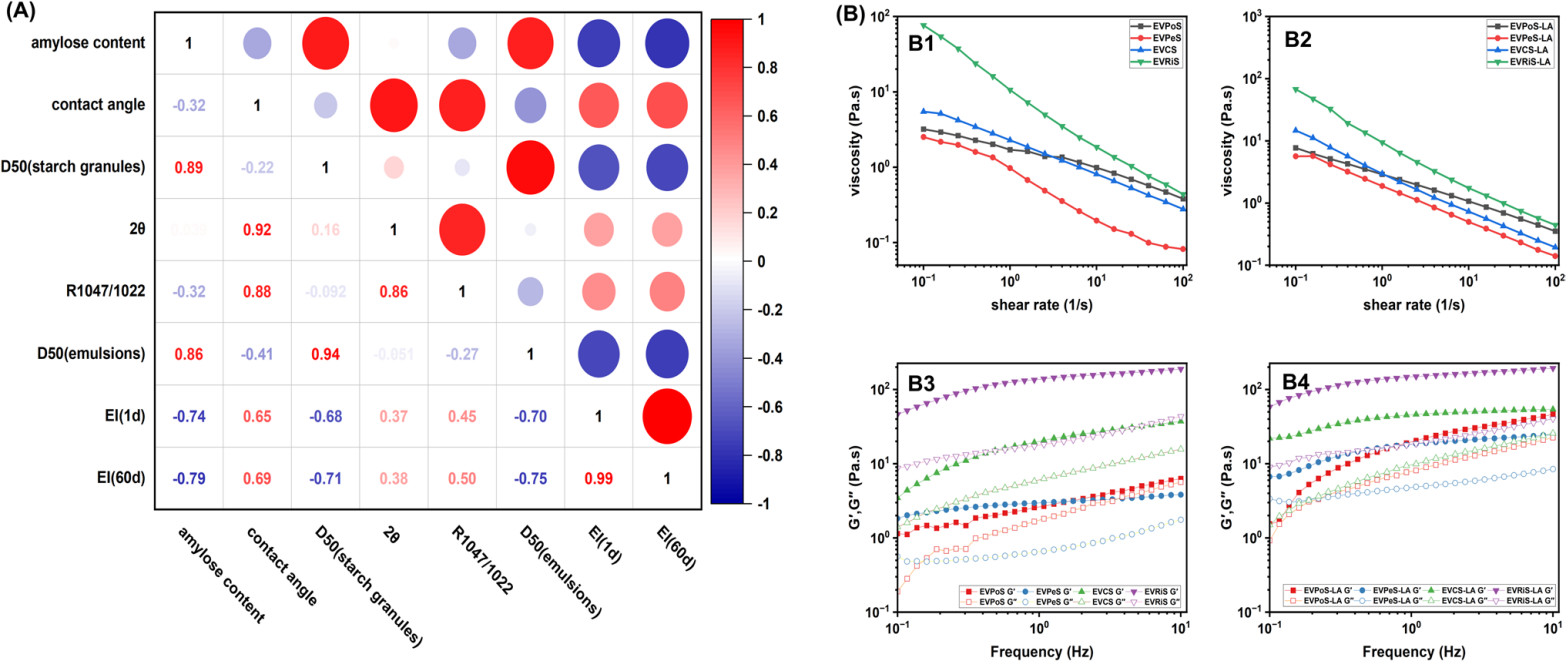
**A** Pearson correlation analysis of EVS and EVS-LA and **B** steady-shear rheological results of Pickering emulsions prepared with EVS and EVS-LA granules, and dynamic viscoelasticity of Pickering emulsions prepared with EVS and EVS-LA granules

The structural–functional relationship between the V-type starch structure and emulsifiability was further illuminated using Pearson correlation coefficients, as illustrated in Fig. 4A. EI exhibited a significant positive correlation with contact angle (*r* = 0.69) and a significant negative correlation with amylose content, D(50) (starch granules), and D(50) (emulsion) (*r* = − 0.79, − 0.71, and − 0.75, respectively). Furthermore, D(50) (emulsion) demonstrated a significant positive correlation with D(50) (starch granules) and amylose content (*r* = 0.94 and 0.86, respectively).

In conclusion, the smaller size of starch granules led to the formation of smaller emulsion droplets in the dispersed phase and higher EI values. The emulsion stabilized by EVRiS-LA particles showed no significant difference in droplet size compared to that stabilized by EVRiS particles. However, storage stability tests revealed striking differences: the EVRiS-LA-stabilized emulsion maintained excellent physical stability (no oiling-off or sedimentation) throughout the 60-day storage period (long-term emulsifiability), whereas the EVRiS-stabilized emulsion exhibited marked destabilization after only 3 days (short-term emulsifiability). The findings suggest that the granule size of V-type starch influences short-term emulsifiability, the wettability and granule size of V-type starch influence long-term emulsifiability.

#### Rheological properties

The rheological properties of Pickering emulsions reflect their stability and functionality. The viscosity of all samples decreased as shear rate increased (Fig. 4B1 and B2), indicating a typical shear-thinning behavior, in line with previous research findings (Zhu, 2019).

It is plausible that EVS and EVS-LA granules have a higher propensity to swell in water. Within emulsions, these granules gradually adopt a linear configuration to form a three-dimensional network structure while still maintaining their particle morphology. Shearing disrupts the entangled polymer network, causing a greater disruption in intermolecular entanglement compared to reformation. As a result, the intermolecular flow resistance decreases, leading to lower apparent viscosity (Chen et al., 2022; Remanan and Zhu, 2023).

Comparatively, the viscosity of EVS-LA samples, except EVRiS-LA, demonstrated an increase. This may be due to the strengthening of hydrophobic interactions among particles, which facilitates the formation of an interconnected network structure. Consequently, when external forces are applied, the Pickering emulsion system exhibits stronger flow resistance. Furthermore, the increased viscosity impedes free movement and hinders fusion, coalescence, and sedimentation, ultimately enhancing the stability of the emulsion (Wang et al., 2023).

The shear stability of the emulsion prepared by EVRiS-LA surpassed that of the other EVS-LA samples, aligning with the sequence EVRiS-LA > EVCS-LA > EVPoS-LA > EVPeS-LA, consistent with the EI results. When the EVS-LA content was fixed at 6.0%, the small starch granules in EVRiS-LA formed a three-dimensional network barrier around the oil droplets (Ge et al., 2017).

Moreover, the *G*′ and *G*ʺ of the emulsions were measured in the low-frequency region of 0.1–10 Hz, as displayed in Fig. 4B3 and B4. At the tested frequencies, all emulsions exhibited higher *G*′ values than *G*ʺ values, suggesting the establishment of an elastic network structure within the O/W emulsion system (Cai et al., 2024a).

The emulsions stabilized by EVS-LA demonstrated higher *G*′ values compared to those stabilized by EVS. This suggests that hydrophobic interactions cause emulsion droplets to tightly associate, forming a denser network. This network restricts the mobility of emulsion droplets, ultimately leading to increased viscosity and modulus values. The trend observed in the *G*′ values of the emulsions aligns well with the changes in average droplet size distribution, which is consistent with previous research findings (Pang et al., 2021).

In conclusion, EVS and EVS-LA were prepared and their structures and physicochemical properties, along with their emulsion properties, were evaluated. Ethanol can induce amylose to form a V-type single-helical structure, and evaporation of the ethanol results endows a hydrophobic cavity to this helical structure. Introducing LA into the cavity structure increased hydrophobicity as shown by contact angle results. Through microscopic observation, both EVS and EVS-LA exhibited rough surfaces. However, starches derived from different botanical sources exhibited significant differences in their physicochemical properties, resulting in varying emulsification effects.

Emulsion properties, including emulsion droplet size and storage stability, were related to the hydrophobicity and particle size of starch. Ethanol precipitation and LA complexation methods had a crucial impact on preparing V-type starch and in enhancing the wettability of starch granules. The granule size of V-type starch influences short-term emulsifiability, the wettability and granule size of V-type starch influence long-term emulsifiability. Similarly, rheological results also indicate that EVS and EVS-LA with smaller particle sizes exhibited stronger viscosity and a network structure. These findings provide profound understanding of the design and development of V-type starch as a multifunctional emulsifier in the food and pharmaceutical industries.

## Supplementary Information

Below is the link to the electronic supplementary material.

Supplementary file1 (DOCX 28036 KB)

Supplementary file2 (DOCX 23 KB)
