# Supplementary material for: V-type granular starches derived from different starch varieties: an exploration of the relationships between structure, physicochemical properties, and emulsifiability
Source: Food Sci Biotechnol. 2025 Jun 2;34(12):2733–47. doi: 10.1007/s10068-025-01898-9 (PMC12240907; doi:10.1007/s10068-025-01898-9)
Supplement: Supplementary file 1 — Supplementary file1 (DOCX 28036 KB) [file 10068_2025_1898_MOESM1_ESM.docx]

**Supplementary Material 2**

**1 Stability of emulsions prepared with EVRiS-LA products**

In the aforementioned experiments, the emulsion prepared using EVRiS-LA particles demonstrated superior emulsifying properties. Consequently, the EVRiS-LA particle emulsion was selected for further stability studies under various conditions.

**1.1 Effect of environmental factors on the stability of Pickering emulsions**

The pH stability of Pickering emulsions was evaluated by adjusting the pH of the EVRiS-LA suspension to 1, 3, 5, 7, 9, and 11 using NaOH or HCl at room temperature. The salt ion stability of Pickering emulsions was evaluated by replacing the aqueous phase with NaCl solutions of different ionic strengths (0, 200, 400, 800, 1200, 2000, 4000, and 8000 mmol/L).

**1.1.2 Zeta-potential measurements**

The Zeta potential of the emulsion was measured using a nanoparticle size and Zeta potential analyzer (Zetasizer Nano ZS 90, Malvern Instruments, UK). Prior to measurement, the Pickering emulsion was diluted 1:10 (v/v) with deionized water and shaken. The measurements were performed at 25 °C and repeated three times for each sample.

**1.1.3 Centrifugal stability measurements**

Weigh 5 g of the emulsion sample and place it in a centrifuge tube. Centrifuge at 837.76 rad/s for 600 s, then take a photograph to record the appearance of the sample (Du et al., 2024).

**1.2 Effect of pH on emulsions stability**

As illustrated in Figure S1 A, no significant changes were observed in the emulsion under different pH conditions. After 60 days of storage, the emulsion remained stable with no oil separation. The EVRiS-LA emulsion displayed a monomodal particle size distribution across various pH levels, with the average particle size decreasing as pH increased, consistent with optical microscopy observations. Centrifugation revealed un-adsorbed particles at the bottom, demonstrating that the emulsion retained emulsifying capacity despite the presence of these particles in the continuous phase.

The absolute value of the Zeta potential of the EVRiS-LA emulsion increased gradually as the pH increased. At pH 11, the emulsion showed a higher absolute Zeta potential, promoting denser particle packing, enhanced adsorption, and reduced desorption at the interface. This formed a dense barrier on the droplet surface, increasing coalescence resistance and improving stability (Tong et al., 2024). In contrast, a lower Zeta potential caused EVRiS-LA particles to aggregate in the aqueous phase, reducing adsorption at the oil-water interface and decreasing emulsion stability.

According to the steric repulsion mechanism, EVRiS-LA particles act as polymer molecules adsorbed at the oil/water interface, generating steric repulsion energy. Thus, the stability of the EVRiS-LA emulsion relies not only on electrostatic repulsion but also on the high molecular weight and network structure of EVRiS-LA, which enhance steric repulsion. This dual mechanism ensures improved emulsion stability under varying conditions.

**1.3 Effect of NaCl concentration on emulsions stability**

As shown in Fig S1 B, the emulsion showed no significant changes under varying salt ion concentrations and remained stable without oil separation after 60 days. Increasing salt ion concentration raised the Zeta potential from approximately −10 mV to around −1 mV, reducing electrostatic repulsion between droplets.

As shown in Fig S1 B, the average particle size of the emulsion increased with salt concentration from 0 to 800 mmol/L, suggesting reduced electrostatic repulsion promoted droplet aggregation. Bimodal particle size distributions observed at 200, 400, and 800 mmol/L further indicate droplet aggregation at higher salt concentrations. Previous studies indicate that high ion concentrations in the aqueous phase reduce electrostatic repulsion between particles, causing their accumulation at the droplet interface and leading to emulsion instability (Lu et al., 2018). Within the salt concentration range of 800-8000 mmol/L, the average particle size of the emulsion decreased progressively as the salt concentration increased.

Centrifugation demonstrated that the emulsion layer height remained consistent across different salt concentrations, with un-adsorbed particle sediment observed at the bottom and no oil separation under the tested conditions. Previous studies have shown that when the zeta potential decreases to the low-charge region, solid particles tend to aggregate, enhancing their network structure in the continuous phase and thereby stabilizing the emulsion (Low et al., 2020). Furthermore, the EVRiS-LA complex provides strong steric hindrance, which reduces van der Waals attraction between droplets and mitigates emulsion instability induced by electrostatic screening.

Consequently, EVRiS-LA-stabilized emulsions demonstrate excellent salt tolerance, making them highly suitable for high-salt food systems.

|  |
| --- |
| Fig. S1 (A) pH stability of Pickering emulsions stabilized by EVRiS-LA particles and (B) salt ion stability of Pickering emulsions stabilized by EVRiS-LA particles. |

Du L, Ru Y, Weng H, Zhang Y, Chen J, Xiao A, Xiao Q. Agar-gelatin Maillard conjugates used for Pickering emulsion stabilization. Carbohydrate Polymers 340: 122293 (2024)

Low LE, Siva SP, Ho YK, Chan ES, Tey BT. Recent advances of characterization techniques for the formation, physical properties and stability of Pickering emulsion. Advances in Colloid and Interface Science 277: 102117 (2020)

Lu X, Zhang H, Li Y, Huang Q. Fabrication of milled cellulose particles-stabilized Pickering emulsions. Food Hydrocolloids 77: 427-435 (2018)

Tong Q, Yi Z, Ma L, Tan Y, Cao X, Liu D, Li X. Influences of carboxymethyl chitosan upon stabilization and gelation of O/W Pickering emulsions in the presence of inorganic salts. Carbohydrate Polymers 331: 121902 (2024)
