# Supplementary material for: V-type granular starches derived from different starch varieties: an exploration of the relationships between structure, physicochemical properties, and emulsifiability
Source: Food Sci Biotechnol. 2025 Jun 2;34(12):2733–47. doi: 10.1007/s10068-025-01898-9 (PMC12240907; doi:10.1007/s10068-025-01898-9)
Supplement: Supplementary file 2 — Supplementary file2 (DOCX 23 KB) [file 10068_2025_1898_MOESM2_ESM.docx]

**Supplementary Material 1**

**Table S1**

The amylose/amylopectin ratio in different starches.

| Sample | amylose/amylopectin ratio |
| --- | --- |
| PoS | 0.6±0.02^b^ |
| PeS | 1.26±0.04^a^ |
| CS | 0.42±0.03^c^ |
| RiS | 0.43±0.01^c^ |

Different lowercase letters (a-c) indicate significant differences (*p* < 0.05).

**Table S2**

Thermal transition parameters of different native starch and V-type starch samples.

| Samples | Peak | | | |
| --- | --- | --- | --- | --- |
|  | *T*_o_ (℃) | *T*_p_ (℃) | *T*_c_ (℃) | Δ*H* (J/g) |
| PoS | 60.4±0.08^c^ | 65.41±0.06^d^ | 77.25±0.29^c^ | 25.51±0.19^a^ |
| PeS | 60.67±0.04^c^ | 70.3±0.08^c^ | 79.83±0.05^b^ | 14.68±0.15^d^ |
| CS | 65.32±0.03^b^ | 71.36±0.01^b^ | 79.57±0.24^b^ | 16.42±0.1^c^ |
| RiS | 69.64±0.18^a^ | 75.8±0.46^a^ | 84.71±0.44^a^ | 17.32±0.1^b^ |
| EVPoS | 47.11±0.38^d^ | 58.74±0.11^e^ | 75.23±0.42^d^ | 7.42±0.36^e^ |
| EVPeS | 47.35±1^d^ | 56.32±0.43^f^ | 71.02±0.9^e^ | 6.5±0.25^f^ |
| EVCS | 46.25±0.36^e^ | 56.48±0.45^f^ | 66.44±0.27^g^ | 4.31±0.29^h^ |
| EVRiS | 44.97±0.13^f^ | 56.1±0.1^f^ | 67.67±0.23^f^ | 5.21±0.05^g^ |

Results are expressed as mean ± standard deviation (*n* = 3). Means with different lowercase letters (a–h) in the same column are significantly different (*p* < 0.05). *T*_o_, onset temperature; *T*_p_, peak temperature; *T*_c_, conclusion temperature; Δ*H*, enthalpy change.

**Table S3**

Thermal transition parameters of different starch-LA complexes.

| Samples | Peak Ⅰ | | | | Peak Ⅱ | | | | Peak Ⅲ | | | |
| --- | --- | --- | --- | --- | --- | --- | --- | --- | --- | --- | --- | --- |
|  | *T*_o_ (℃) | *T*_p_ (℃) | *T*_c_ (℃) | Δ*H*_1_ (J/g) | *T*_o_ (℃) | *T*_p_ (℃) | *T*_c_ (℃) | Δ*H*_2_ (J/g) | *T*_o_ (℃) | *T*_p_ (℃) | *T*_c_ (℃) | Δ*H*_3_ (J/g) |
| LA | 42.6±0.02^b^ | 46.8±0.39^a^ | 50.3±0.66^a^ | 258.11±3.73^a^ | ⸺ | ⸺ | ⸺ | ⸺ | ⸺ | ⸺ | ⸺ | ⸺ |
| EVPoS-LA | 42.72±0.09^a^ | 44.41±0.13^b^ | 47.3±0.32^b^ | 19.95±0.2^b^ | 95.99±1.34^a^ | 101.6±0.07^b^ | 106.43±0.06^b^ | 3.32±0.36^c^ | 116.88±1.59^c^ | 123.15±0.31^c^ | 128.69±1.31^c^ | 2.56±0.3^c^ |
| EVPeS-LA | 42.71±0.04^a^ | 43.71±0.1^c^ | 46.11±0.02^c^ | 5.93±0.02^d^ | 88.65±0.59^c^ | 97.53±0.11^d^ | 105.12±0.67^c^ | 2.82±0.08^d^ | 119.73±0.05^b^ | 130.96±0^a^ | 137.83±0.03^a^ | 7.59±0.34^a^ |
| EVCS-LA | 42.52±0.05^b^ | 43.81±0.02^c^ | 46.39±0.11^c^ | 12.34±0.18^c^ | 90.41±0.43^b^ | 102.31±0^a^ | 108.91±0.02^a^ | 6.37±0.28^a^ | 122.31±0.04^a^ | 124.45±0.18^b^ | 129.9±0.74^c^ | 3.2±0.85^c^ |
| EVRiS-LA | 42.47±0.01^bc^ | 43.75±0.04^c^ | 46.37±0.18^c^ | 13.51±0.27^c^ | 91.33±0.26^b^ | 100.69±0.16^c^ | 106.66±0.15^b^ | 3.78±0.15^b^ | 116.97±0.33^c^ | 124.35±1.28^bc^ | 132.59±1.78^b^ | 5.48±0.27^b^ |

Data are represented as mean ± SD. Means with different lowercase letters (a-d) in the same column are significantly different (p < 0.05). *T_o_*, onset temperature; *T_p_*, peak temperature; *T_c_*, conclusion temperature; Δ*H*, enthalpy change.
